# Sonographic evaluation of median nerve cross‐sectional area in a normal Iranian population: A cross‐sectional study

**DOI:** 10.1002/hsr2.1393

**Published:** 2023-06-29

**Authors:** Seyed Mansoor Rayegani, Masume Bayat

**Affiliations:** ^1^ Physical Medicine and Rehabilitation Research Center Shahid Beheshti University of Medical Sciences Tehran Iran

**Keywords:** cross‐sectional area, median nerve, reference values, ultrasonography

## Abstract

**Introduction:**

Considering disagreements on the normal range of median nerve cross‐sectional area (MNCSA) and insufficient data in the Iranian population, this study aimed to measure normal MNCSA.

**Methods:**

In this cross‐sectional study, bilateral upper limbs of 99 subjects were assessed by sonography, and MNCSA was measured at three levels: forearm, carpal tunnel inlet (CTI), and carpal tunnel outlet (CTO). The association between MNCSA and demographic factors was assessed.

**Results:**

Mean MNCSA was 6.33 mm^2^ at the forearm, 9.41 mm^2^ at CTI, and 10.67 mm^2^ at CTO. MNCSA was significantly higher in males (6.78 vs. 5.94 mm^2^ at the forearm, 9.98 vs. 8.92 mm^2^ at CTI, and 11.24 vs. 10.84 mm^2^ at CTO in males and females, respectively) and taller (>170 cm) subjects in all three levels (6.69 vs. 6.03 mm^2^ at the forearm, 9.80 vs. 9.02 mm^2^ at CTI, and 11.27 vs. 10.12 mm^2^ at CTO in taller and shorter subjects, respectively). MNCSA was not significantly associated with wrist ratio (WR) or body mass index (BMI).

**Conclusion:**

The normal MNCSA range in the Iranian population is 6.31 mm^2^ (forearm) to 10.74 mm^2^ (CTO). MNCSA is significantly higher in males and taller subjects but is not associated with BMI and WR.

## 1 INTRODUCTION

The carpal tunnel or carpal canal is a bony‐ligamentous structure in the palmar wrist area that is formed by the wrist bones dorsally and flexor retinaculum ventrally. It connects the forearm to the hand and surrounds the long flexor tendons in addition to the median and ulnar nerves. The canal is a narrow structure; thus, even small decreases in space or increases in pressure can result in the entrapment of the median nerve and, ultimately, the occurrence of carpal tunnel syndrome (CTS).

CTS affects about 9.2% of women and 6% of men.^1^ The diagnosis of CTS is based on history and physical examination and confirmed by electrodiagnostic studies. Due to high sensitivity (84%) and specificity (95%), electrodiagnosis is the gold‐standard diagnostic tool.^2^ In recent years, ultrasonography has gained popularity for musculoskeletal assessments. Specifically, sonographic evaluation of the median nerve at the wrist to detect CTS is noteworthy. High‐resolution images, noninvasiveness, and being less time‐consuming are advantages of this method over electrodiagnosis. Although sonographic criteria have not been fully determined for CTS, some sonographic findings favor this diagnosis.

Among the several indicators, increased median nerve cross‐sectional area (MNCSA) is the most significant and possibly most reliable finding.^3^ MNCSA can be measured in different locations, including the forearm, the carpal tunnel inlet (CTI) (at the level of pisiform), and the carpal tunnel outlet (CTO). Among these, MNCSA at the CTI level is more frequently studied and is increased in CTS. A study comparing sonography with electrodiagnosis demonstrated that both methods have the same sensitivity for diagnosing CTS.^4^ However, another study compared the results of MNCSA measurement with electrodiagnosis and found that sonography is a fairly sensitive method for diagnosing CTS but still not competent enough to replace electrodiagnosis.^5^

There are several studies with different results for a normal range of MNCSA. In the most recent review, the mean MNCSA was 8.81 mm^2^ at mid‐arm, 8.57 mm^2^ at the elbow, 7.07 mm^2^ at mid‐forearm, 8.74 mm^2^ at CTI, and 9.02 mm^2^ at CTO.^6^ Another study reported normal MNCSAs to be 8.2 mm^2^ at the level of the radiocarpal joint, 8.3 mm^2^ at CTI, and 8.1 mm^2^ at CTO.^7^

A handful of studies have provided reference values for MNCSA in certain sample populations.^8^, ^9^ However, such studies have yet to be widely used. Furthermore, they are often limited to a specific population and may not be useful for all future research.

Although studies on normal range of MNCSA have demonstrated differences among various populations, to the best of our knowledge, it has not yet been studied in the Iranian population. This study was designed to measure normal MNCSA in the Iranian population, as and to evaluate its association with sex, body mass index (BMI), wrist ratio (WR), and height.

## 2 PATIENTS AND METHODS

This cross‐sectional descriptive study was performed on healthy individuals who presented to the university hospitals. The university ethics committee approved this study, and the research was conducted in accordance with the Declaration of the World Medical Association. Ninety‐nine healthy subjects (46 men and 53 women) were enrolled in the study. All participants provided written informed consent. Participants were recruited from healthy subjects with no signs or symptoms of median nerve compromise, including numbness, tingling, burning pain, paresthesia, or hypoesthesia in the median innervation parts of the hand. Inclusion criteria comprised age from 18 to 70 years and absence of diseases or conditions predisposing to median neuropathy like thyroid diseases and steroid use. Exclusion criteria included the presence of CTS signs or symptoms, history of diabetes or rheumatological diseases, previous wrist fracture, pregnancy, or cervical radiculopathy. Demographic characteristics, including age, sex, height, weight, BMI, and WR, were obtained. To measure WR, we calculated the ratio of anteroposterior to mediolateral diameters. These parameters were measured at the distal wrist crease using a standard sliding caliper. General observations suggest that a 0.70 WR may be the critical point at which latencies tend to reach the upper limits of normal.^10^ This was set as the cut‐off point for the present study. To assess height, WR, and BMI, participants were categorized into two groups of >170 and ≤170 cm, WRs >0.70 and ≤0.70, and BMIs >25 and ≤25, and the MNCSA was compared between the two groups.

**Figure 1:**
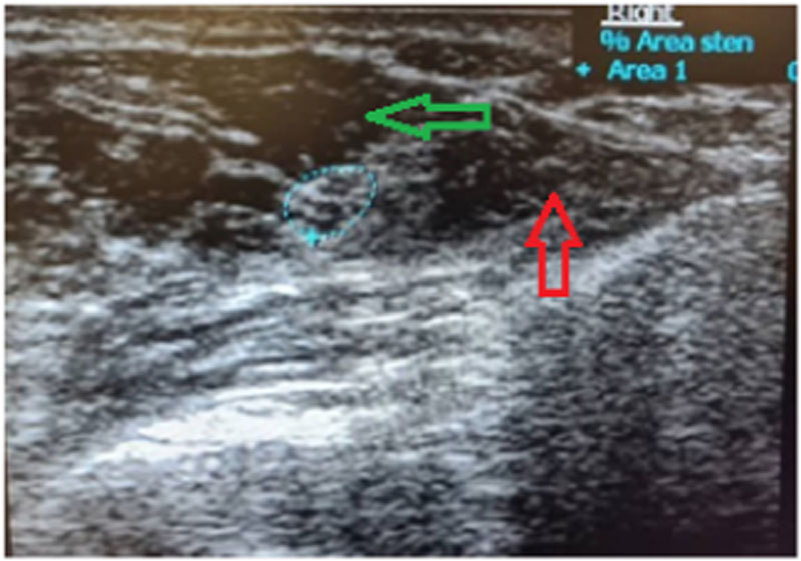
MNCSA measurement at the forearm. Blue dotted line: Median nerve; Red arrow: Flexor Digitrum Profondus muscle; Green arrow: Flexor Difgitrum Superficialis muscle. MNSCA, median nerve cross‐sectional area.

**Figure 2:**
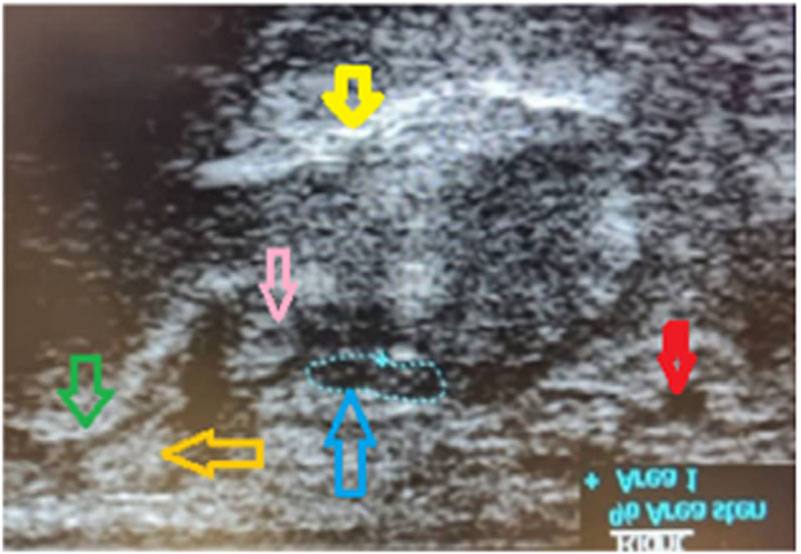
MNCSA measurement at carpal tunnel inlet. Blue dotted line: Median nerve; Red arrow: Ulnar artery; Yellow arrow: Lunate bone; Pink arrow Flexor Pollicis Longus tendon; Orange arrow: Flexor Carpi Radialis tendon; Green arrow: Scaphoid bone. MNSCA, median nerve cross‐sectional area.

**Figure 3:**
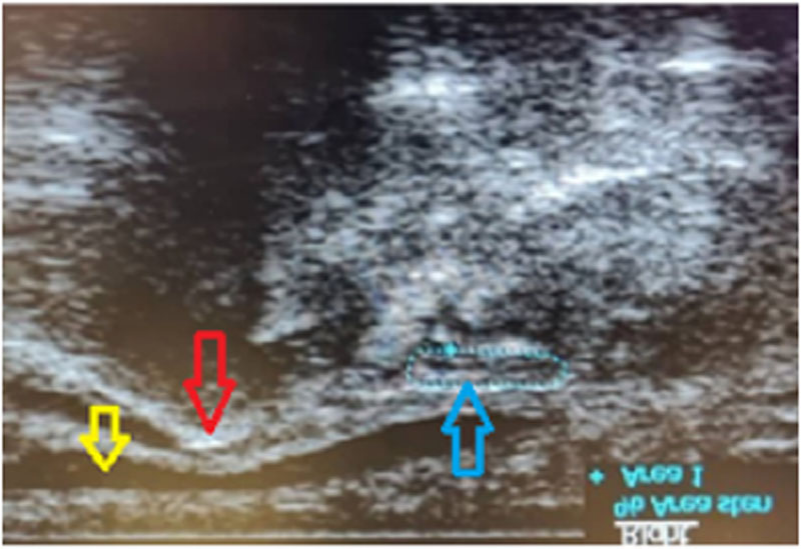
MNCSA measurement at carpal tunnel outlet. Blue dotted line: Median nerve; Red arrow: Trapezium bone; Yellow arrow: the Thenar muscles. MNSCA, median nerve cross‐sectional area.

Both upper limbs in all participants were evaluated by sonography, and MNCSA was measured at the forearm (Figure 1), CTI (Figure 2), and CTO (Figure 3). Sonographic imaging was performed by a physical medicine and rehabilitation professor who has undergone educational courses in limb ultrasonography and has performed more than 700 cases of musculoskeletal ultrasonography per year in a matter of 8 years. Also, we assessed the intra‐observer reliability of the measurements. MNCSA was measured three times in each area, and the mean of the three was used as the final measurement. The ultrasound device was a Philips HD6 machine with a 3–12 MH linear probe. To obtain images, subjects sat in front of the practitioner with the forearm in supination, the wrist in the neutral position, and the fingers semi‐flexed. To detect the median nerve, the probe was positioned on the forearm (about 7 cm above the wrist crease) in a transverse orientation. Anatomical and sonographic landmarks of carpal tunnel are the lunate and capitate bones on the floor, and four bony elements limit the sides: pisiform and scaphoid tubercle proximally, and the hook of hamate and trapezius tubercle distally. To measure MNCSA, the nerve margin was tracked by the sonography caliper. The nerve margin was detected as the intersection of hypoechoic nerve fascicles and hyperechoic nerve sheaths. Each measurement was repeated three times, and mean values were recorded as the final result.

### 2.1 Statistical analyses

Data were analyzed using SPSS version 25 for windows. The quantitative variables like age and the clinical outcomes were expressed as mean ± SD, and the categorical variables like sex and patient's group were expressed as frequency and percentage. The Mann–Whitney *U* test was used to compare the outcomes between the groups. Intra‐observer reliability was assessed by intraclass correlation coefficients based on the 95% confidence interval for absolute agreement. A correlation coefficient of 0–0.20 indicated poor reliability. A correlation coefficient of 0.21–0.40 indicated fair reliability. Those from 0.41 to 0.60 indicated moderate reliability. Those from 0.61 to 0.80 indicated good reliability, and a correlation coefficient greater than 0.81 indicated excellent reliability. *p* Values < 0.05 were considered statistically significant.

## 3 RESULTS

The data for 198 hands from 99 participants (46 men and 53 women) were analyzed. The mean age of subjects was 41 years (19–63). The mean WR was 0.67, and the mean BMI was 25.6 (Table 1). The mean MNCSA was 6.33 mm^2^ in the forearm (ranging from 3.0 to 9.8 mm^2^), 9.41 mm^2^ in CTI (ranging from 4.4 to 14.0 mm^2^), and 10.67 mm^2^ in CTO (ranging from 5.3 to 16.2 mm^2^; Table 2).

**Table 1:** Characteristics of participants.

	Mean (SD)	Range
Age	41.7 (13.3)	19–63
BMI	25.6 (4.0)	16.6–37.4
WR	0.67 (0.4)	0.63–0.71

Abbreviations: BMI, body mass index; WR, wrist ratio.

**Table 2:** Mean MNCSA at three different levels.

Level of measurement	Mean (SD)		Range	
	Right	Left	Right	Left
Forearm	6.31 (1.35)	6.36 (1.39)	3.30–9.10	3.00–9.80
CTI	9.29 (2.27)	9.54 (1.68)	4.41–14.00	5.90–13.80
CTO	10.60 (1.89)	10.74 (2.05)	6.80–16.20	5.30–16.20

Abbreviations: CTI, carpal tunnel inlet; CTO, carpal tunnel outlet; MNCSA, median nerve cross‐sectional area.

Sex distribution included 53 females and 46 males. MNCSA was significantly higher in men (6.78 vs. 5.94 mm^2^ at the forearm, 9.98 vs. 8.92 mm^2^ at CTI, and 11.24 vs. 10.84 mm^2^ at CTO for males and females, respectively; Table 3).

**Table 3:** Differences in sex, height, WR, and BMI of MNCSA.

	Level of measurement								
	Forearm			CTI			CTO		
Variable	Right	Left	*p* Value	Right	Left	*p* Value	Right	Left	*p* Value
**Sex**									
Male	6.82 (1.23)	6.75 (1.36)	0.001	9.95 (1.83)	10.02 (1.60)	0.007	11.22 (1.82)	11.27 (1.75)	0.002
Female	5.87 (1.30)	6.02 (1.35)	0.009	8.73 (2.47)	9.11 (1.62)	0.007	10.06 (1.80)	10.28 (2.18)	0.016
**Height**									
>170 cm	6.69 (1.34)	6.69 (1.37)	0.02	9.74 (2.25)	9.86 (1.70)	0.04	11.20 (1.77)	11.35 (1.68)	0.005
≤170 cm	5.99 (1.30)	6.07 (1.38)	0.01	8.85 (2.21)	9.20 (1.59)	0.05	10.05 (1.86)	10.19 (2.22)	0.003
**WR**									
>0.7	6.39 (1.36)	6.54 (1.44)	0.61	9.35 (2.31)	9.69 (1.60)	0.38	10.85 (1.90)	10.92 (2.06)	0.24
≤0.7	6.25 (1.35)	6.21 (1.35)	0.25	9.25 (2.26)	9.41 (1.75)	0.40	10.40 (1.87)	10.60 (2.04)	0.43
**BMI**									
>25	6.12 (1.47)	6.62 (1.24)	0.06	9.40 (2.19)	9.76 (1.39)	0.19	10.69 (1.73)	11.00 (1.81)	0.22
≤25	6.12 (1.47)	6.10 (1.49)	0.88	9.19 (2.36)	9.32 (1.90)	0.64	10.51 (2.04)	10.50 (2.23)	0.88

*Note*: Data presented as mean (SD).

Abbreviations: BMI, body mass index; CTI, carpal tunnel inlet; CTO, carpal tunnel outlet; MNSCA, median nerve cross‐sectional area; WR, wrist ratio.

The mean MNCSA was significantly different between height groups and at all levels. The results showed that taller subjects had a larger MNCSA (6.69 vs. 6.03 mm^2^ at the forearm, 9.80 vs. 9.02 mm^2^ at CTI, and 11.27 vs. 10.12 mm^2^ at CTO for >170 cm and ≤170 cm subjects, respectively; Table 3).

WR was measured in all subjects, and mean values were 0.68 among males and 0.66 among females, which was not significantly different. Also, no significant difference was observed between the MNCSA of the WR groups (6.45 vs. 6.23 mm^2^ at the forearm, 9.52 vs. 9.33 mm^2^ at CTI, and 10.88 vs. 10.50 mm^2^ at CTO for WR >0.7 and ≤0.7, respectively; Table 3).

The mean BMI was 26.5 for men and 24.8 for women. No significant difference was observed in the MNCSA of the two BMI groups (6.37 vs. 6.11 mm^2^ at the forearm, 9.58 vs. 9.25 mm^2^ at CTI, and 10.84 vs. 10.50 mm^2^ at CTO for BMIs >25 and ≤25, respectively; Table 3).

The measurements had good intra‐observer reliability (Table 4).

**Table 4:** Intra‐observer reliability of MNCSA measurements.

Level of measurement	Observe 1 and 2		Observe 1 and 3		Observe 2 and 3	
	Coefficient	*p* Value	Coefficient	*p* Value	Coefficient	*p* Value
Forearm	0.99	0.001	0.98	0.001	0.98	0.001
CTI	0.98	0.001	0.97	0.001	0.99	0.001
CTO	0.76	0.001	0.74	0.001	0.98	0.001

Abbreviations: CTI, carpal tunnel inlet; CTO, carpal tunnel outlet; MNSCA, median nerve cross‐sectional area.

## 4 DISCUSSION

The clinical significance of measuring MNCSA is in detecting CTS. Inflammation and secondary edema happen after an increased pressure in the canal, which leads to nerve enlargement, which is observed as increased MNCSA. This enlargement is especially more obvious in the early stages of CTS when nerve atrophy has not yet occurred. The point of disputation lies in the normal range of MNCSA and the cutoff point for the diagnosis of CTS. No consensus exists on the normal upper limit of MNCSA. Currently, a broad range of 8.5–15 mm^2^ is considered the normal range of MNCSA.^11^, ^12^

In this cross‐sectional descriptive study on 99 healthy participants, bilateral MNCSA was measured at three levels: forearm, CTI, and CTO. MNCSA ranged from 6.31 mm^2^ at the forearm to 10.74 mm^2^ at CTO. Although normal upper limits were not measured, the present study found mean values to be 9.41 mm^2^ at CTI and 10.67 mm^2^ at CTO. Kang et al. determined forearm MNCSA to be 6.8 mm^2^, which is very close to the findings of this study.^13^ In another study, the mean value of wrist MNCSA was estimated to be 8.5–11 mm^2^, which is also close to this study's findings.^14^ Bathala et al. found the mean MNCSA to be 7.2 mm^2^ at the wrist and 4.8 mm^2^ at the mid‐forearm level, which is significantly smaller than the findings of this study.^15^ The variety in normal values can be due to differences in age, weight, height, sex, and ethnicity.^16^, ^17^, ^18^, ^19^

Regarding ethnicity, Ng et al. in a systematic review observed that the mean CSA of median nerve appears to be the smallest at elbow in Indian population (6.90 mm^2^). At mid‐forearm and CTI, the values are similar: the mean CSA of median nerve is higher in Caucasian (8.20 mm^2^) compared to the Indians (5.64 mm^2^) and Chinese (5.70 mm^2^) subjects. At CTI, the Caucasian (8.50 mm^2^), Japanese (8.50 mm^2^), and Chinese (8.12 mm^2^) CSA values were similar. The median nerve mean CSA at CTO (8.97 mm^2^) was available for Chinese subjects only. These findings suggest the differences in median nerve CSA for different ethnicities.^6^

The impact of these factors has been mentioned in several studies,^15^, ^16^, ^20^, ^21^, ^22^, ^23^, ^24^ and some have been evaluated in the present study. Bae et al. found that CSA is significantly correlated with gender, height, weight, and BMI.^20^ Considering sex differences, this study's findings indicated a significant difference between MNCSA in females and males. According to the results, MNCSA was significantly higher in male participants. Kang et al. also demonstrated MNCSA to be significantly higher in males.^21^ In a study on CSA of the sciatic nerve, women had smaller CSA than men.^18^ The findings of this study are consistent with previous research.

A significant association between height and MNCSA has been demonstrated in this study. In their study on the sciatic nerve, Singh et al. found that MNCSA was positively correlated with height, weight, and BMI.^16^ However, in a study by Higginbotham et al., the correlation with height was not significant.^22^ This discordance can be due to the small sample size (21 cases) in Higginbotham's study. In the current study, no significant association was observed between BMI and MNCSA. There are controversies regarding this association. Some studies emphasize that MNCSA is influenced by BMI.^21^ On the other hand, Higginbotham et al. found no significant correlation between CSA and BMI.^22^

A meta‐analysis by Shiri et al. suggests that a square‐shaped wrist predicts CTS in both men and women.^25^ In a study by Thiese et al., a square wrist was significantly associated with CTS after controlling for confounders, including BMI.^26^ Another study by Palve et al. concluded that WR has a progressive correlation with CTS severity, but statistical significance was seen only in moderate and severe CTS.^27^ On the other hand, several other studies did not confirm a significant relationship or showed only a weak association.^28^, ^29^ In the present study, no significant association was observed between WR and MNCSA. The small sample size and inadequate control of confounding factors such as BMI and occupational exposures have limited analysis and power.

There are questions regarding the best anatomic location for diagnostic CSA measurement. It is generally accepted that one of the most important parameters for detecting CTS is an increase in MNCSA at CTI.^30^ On the other hand, some authors advocate measuring MNCSA at CTO.^31^ In the present study, the largest diameter was observed at CTO. Evidently, MNCSA has its largest diameter at the level of the hamate bone (CTO).^6^

The present study had some limitations. For one, this study aimed to determine the normal values of MNCSA in the Iranian population, which needs a bigger and more heterogeneous sample size. Another limitation was the reliance on subjective history for participant selection. For a more definite diagnosis or rule out of CTS, it would be more favorable to use objective tests, such as questionnaires or electrodiagnostic studies. However, earlier studies reported good agreement with MNCSA measurements.^32^ A great strength of this study was evaluating a normal MNCSA in a sample of the Iranian population and evaluating the impact of BMI, sex, and WR. To the best of our knowledge, no similar study has been performed on the Iranian people. All previous studies have compared sonography with electrodiagnosis in CTS cases or evaluated the use of MNCSA for CTS grading.^5^, ^33^, ^34^, ^35^ Another strength of this study was evaluating MNCSA at three levels and assessing its association with multiple factors. However, this study did not consider the effect of other factors, such as a job or sports activities, that potentially impact MNCSA.

Based on the present study's findings, the normal range of MNCSA in the Iranian population is 6.31 mm^2^ (at forearm) to 10.74 mm^2^ (at CTO), which is similar to the results of other studies. MNCSA is significantly higher in males and taller subjects, but there is no significant association between MNCSA and BMI or WR. Reaching a more precise conclusion demands further studies with larger sample sizes.

## AUTHOR CONTRIBUTIONS

**Seyed Mansoor Rayegani**: Conceptualization; investigation; methodology; resources; supervision. **Masume Bayat**: Data curation; investigation; project administration; writing—original draft; writing—review and editing.

## CONFLICT OF INTEREST STATEMENT

The authors declare no conflict of interest.

## TRANSPARENCY STATEMENT

The lead author Masume Bayat affirms that this manuscript is an honest, accurate, and transparent account of the study being reported; that no important aspects of the study have been omitted; and that any discrepancies from the study as planned (and, if relevant, registered) have been explained.

## Data Availability

The data that support the findings of this study are available from the corresponding author upon reasonable request.
